# Patterns of Bruton’s Tyrosine Kinase Inhibitor (BTKi) Usage in B-cell Lymphomas in India: A Questionnaire-Based Study

**DOI:** 10.7759/cureus.99794

**Published:** 2025-12-21

**Authors:** Azra Naseem, Amullya Pednekar, Saiprasad Patil, Hanmant Barkate

**Affiliations:** 1 Global Medical Affairs, Glenmark Pharmaceuticals Limited, Mumbai, IND

**Keywords:** b-cell lymphomas, bruton’s tyrosine kinase inhibitors, chronic lymphocytic leukemia, cytogenetic markers, molecular testing

## Abstract

Purpose: This cross-sectional questionnaire-based survey assessed the use of Bruton’s tyrosine kinase inhibitors (BTKis), clinical decision-making, and barriers to implementation among Indian hematologists managing B-cell lymphomas, identifying practice gaps that require policy interventions.

Materials and methods: A 28-item structured questionnaire was administered to hematologist-oncologists across India from 1 February 2025 to 31 May 2025. Of 50 invited clinicians, 38 responded (76% response rate). The survey assessed BTKi usage by disease and therapy line, molecular testing patterns, drug selection strategies, adverse event management, discontinuation reasons, and prescribing barriers. Data were analyzed using descriptive statistics with Microsoft Excel (Microsoft Corp., Redmond, WA, USA).

Results: Diffuse large B-cell lymphoma (40%) and chronic lymphocytic leukemia (CLL, 22%) were the most common. BTKis were used as first-line therapy in 92.1% of CLL cases. Generic acalabrutinib showed higher adoption (42.1%) than ibrutinib, mainly due to better safety and cost advantages. Cytogenetic markers were the most influential factor in initiating BTKi (52.63%). Molecular testing adoption exceeded 63%, with TP53, del(17p), and IgHV routinely ordered. For elderly or frail patients, 86.8% preferred BTKis over chemotherapy. Intolerance and financial constraints were the leading causes of discontinuation (36.8% and 34.2%). High cost was the significant barrier (89.47%), followed by side-effect management (42.1%) and drug availability (36.84%). Over 86% anticipate increased use of first-line BTKis within five years.

Conclusions: This survey reveals evidence-based treatment selection and appropriate risk stratification in Indian hematology practice. The predominance of generic acalabrutinib reflects superior safety compared to ibrutinib and cost considerations. Financial constraints driving treatment discontinuation underscore an urgent need for healthcare policy interventions to improve drug accessibility and affordability, ensuring equitable access and optimal outcomes.

## Introduction

Bruton’s tyrosine kinase (BTK) occupies a central role in the signaling cascade of the B-cell receptor (BCR), orchestrating key steps in B-cell development, activation, and survival. Activation of the BCR triggers downstream molecular events, with BTK functioning as a mediator that activates PLCγ2 and other effectors essential for proliferation and resistance to apoptosis [1-3]. Aberrant or chronic BCR signaling underlies numerous B-cell lymphoid malignancies, making BTK inhibition a rational and transformative therapeutic strategy [4,5].

The advent of oral BTK inhibitors (BTKis) has revolutionized the management of these diseases over the past decade [6,7]. Ibrutinib, the first covalent BTKi, was approved in 2013 for relapsed/refractory mantle cell lymphoma (MCL) and later for chronic lymphocytic leukemia (CLL), ushering in a shift toward chemotherapy-free regimens. It markedly improved outcomes, including in high-risk CLL with del(17p) or TP53 mutations, and demonstrated strong efficacy in other B-cell malignancies such as Waldenström macroglobulinemia, with response rates approaching 90% [8-10], and marginal zone lymphoma (MZL) [11]. The initial approvals were later revoked [12]; however, subsequent clinical evidence supported expanded approvals of other agents for these indications.

Despite the remarkable clinical benefits, ibrutinib’s off-target kinase inhibition (e.g., EGFR, TEC, ITK) is associated with adverse effects, including atrial fibrillation and a bleeding tendency. This prompted the development of second-generation covalent BTKis, such as acalabrutinib and zanubrutinib, with greater selectivity [13-16]. These agents have shown comparable efficacy to ibrutinib with improved tolerability; for example, in a head-to-head trial in previously treated CLL, acalabrutinib achieved non-inferior progression-free survival to ibrutinib with significantly lower rates of atrial fibrillation and fewer discontinuations due to adverse events [17]. Zanubrutinib also demonstrated a better safety profile and a superior overall response rate (94.5%) in specific settings (such as relapsed CLL) compared with ibrutinib [18]. More recently, the emergence of non-covalent (reversible) BTKis, such as pirtobrutinib, offers promising options for patients who develop resistance to covalent drugs. Pirtobrutinib has shown high response rates even in heavily pretreated CLL and MCL patients after prior BTKi failure, while exhibiting a markedly lower incidence of BTKi-class toxicities [19,20].

Globally, BTKis are now firmly established as the standard of care for multiple B-cell lymphomas [13]. In resource-rich settings, uptake of the second-generation BTKis acalabrutinib and zanubrutinib has been rapid and substantial, fundamentally transforming treatment paradigms for B-cell malignancies [21]. However, applying these breakthroughs in lower-middle-income countries poses unique challenges. Conventional chemoimmunotherapy (e.g., chlorambucil- and bendamustine-based regimens) remains in use longer here primarily due to cost considerations [22-24]. In the Indian setting, only patients who can afford out-of-pocket expenses or have adequate insurance coverage can access novel agents in line with international standards. In contrast, others often face treatment delays or modifications due to financial constraints [25].

There is a paucity of published data on how Indian clinicians are integrating BTKis into routine practice amid these constraints. Understanding real-world usage patterns is essential for identifying gaps in care and informing future strategies, including targeted educational initiatives and policy interventions to improve access. Accordingly, we conducted a questionnaire-based survey of hematologists across India to assess current BTKi use in B-cell malignancies systematically. This first national survey describes practice patterns related to BTKi selection and combinations, biomarker testing, toxicity management, and implementation barriers, and contextualizes these findings within global practices and evolving treatment paradigms.

## Materials and methods

This study was conducted as a cross-sectional, questionnaire-based survey to describe real-world patterns of BTKi use among hematologists in India. The primary objective was to characterize current BTKi prescribing practices in B-cell malignancies. In contrast, secondary objectives included evaluating factors influencing treatment selection, biomarker testing practices, toxicity management, and barriers to BTKi implementation in routine clinical practice.

The survey was conducted over four months, from 1 February 2025 to 31 May 2025. The target population comprised consultant hematologist-oncologists involved in the management of B-cell lymphomas and leukemias. Participants were identified using a convenience sampling approach through professional networks, national hematology/oncology society membership directories, and peer referrals. An effort was made to include representation from different geographic regions and practice types (academic tertiary centers and private practice groups) to capture a broad perspective of national practices.

The study was approved by the Healthcare Ethics Committee, Ghaziabad, Uttar Pradesh, India (approval number: NIS/2025/30). Participation was voluntary, electronic informed consent was obtained prior to survey initiation, and all responses were anonymized.

A structured, study-specific questionnaire comprising 28 items was developed (Appendices). The questionnaire included multiple-choice, Likert-scale, and limited open-text questions covering demographics, BTKi use by indication and line of therapy, biomarker testing practices, adverse event management, reasons for treatment discontinuation, and perceived barriers to BTKi use. “Clinically fit” patients were defined as those with good performance status and limited comorbidities. Content validity was established through review by an expert panel of five senior hematologists, and revisions were made based on their feedback. The expert panel consisted of hematologists with more than 15 years of experience working at a tertiary medical center.

The survey was administered in English using SurveyMonkey® (SurveyMonkey Inc., San Mateo, CA, USA). Personalized, single-use survey links were distributed via email and professional messaging platforms. Two reminder messages were sent to non-responders at one-week intervals. Of the 50 hematologists invited, 38 responded (response rate: 76%). Responses were checked for completeness, and a duplication check was performed on the entire dataset. All 38 responses were fully completed (100% item completion) and were therefore considered valid for analysis. No formal sample size calculation was performed because the study was exploratory and descriptive.

Data were analyzed using descriptive statistics in Microsoft Excel (Microsoft Corp., Redmond, WA, USA). Categorical variables were summarized as frequencies and percentages. No hypothesis testing or comparative analyses were performed. Results are presented primarily as proportions of respondents and visualized using bar and pie charts. The study was designed and reported in accordance with the Checklist for Reporting Results of Internet E-Surveys (CHERRIES) guidelines.

## Results

Demographics and clinical experience

Of the 50 hematologists invited, 38 responded to the survey, yielding a response rate of 76%. The distribution of respondents by years of clinical experience is summarized in Table 1.

**Table 1:** Respondent demographics (years of clinical experience)

Years of experience (years)	n (%)
0-5	3 (7.89)
6-10	12 (31.58)
11-15	7 (18.42)
15-20	11 (28.95)
>20	5 (13.16)

B-cell lymphoma disease patterns

The survey revealed important insights into the prevalence of different B-cell lymphomas in Indian hematology practice. Diffuse large B-cell lymphoma (DLBCL) emerged as the most commonly encountered malignancy, accounting for an average of 40% of cases across responding practices. CLL represented 22% of cases, followed by follicular lymphoma (FL) at 13%, MCL at 8%, MZL at 6%, and Waldenström’s macroglobulinemia (WM) at 5%.

Clinical presentation patterns

**Figure 1:**
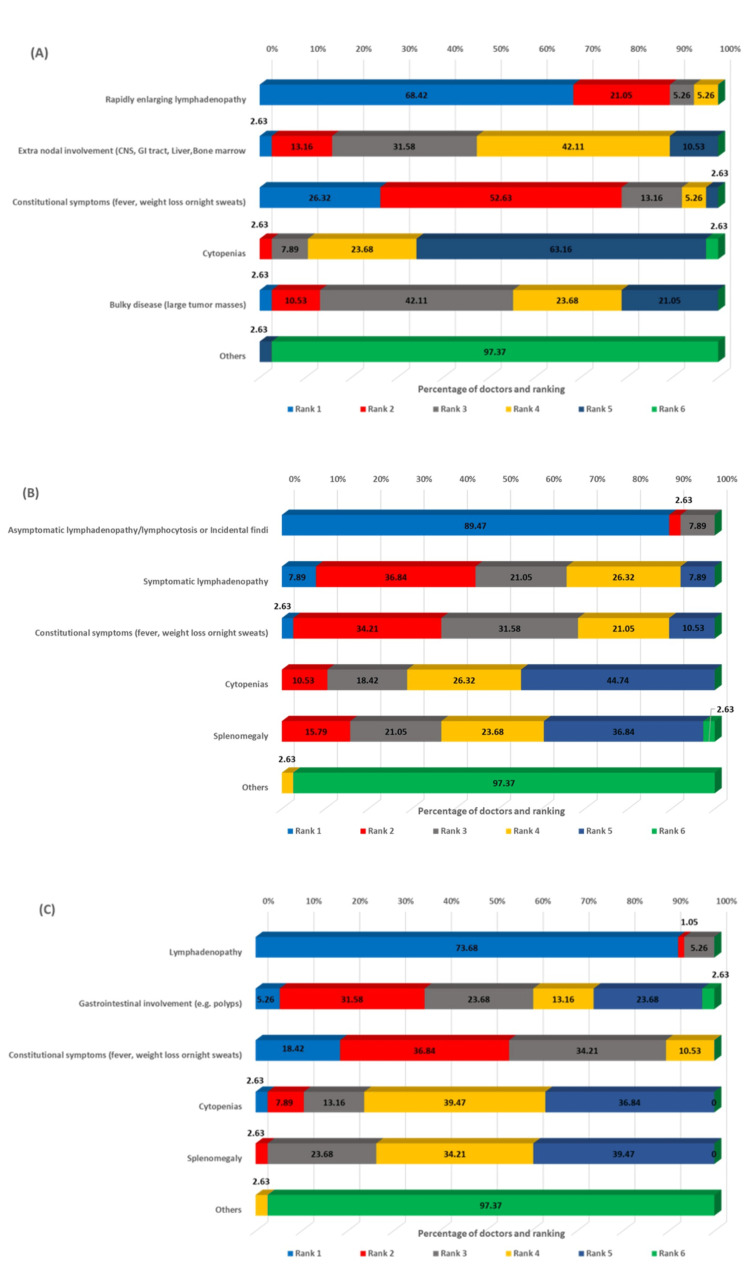
Ranking of clinical presentations from most common to least common in DLBCL (A), CLL (B), and MCL (C) DLBCL: diffuse large B-cell lymphoma, CLL: chronic lymphocytic leukemia, MCL: mantle cell lymphoma

**Figure 2:**
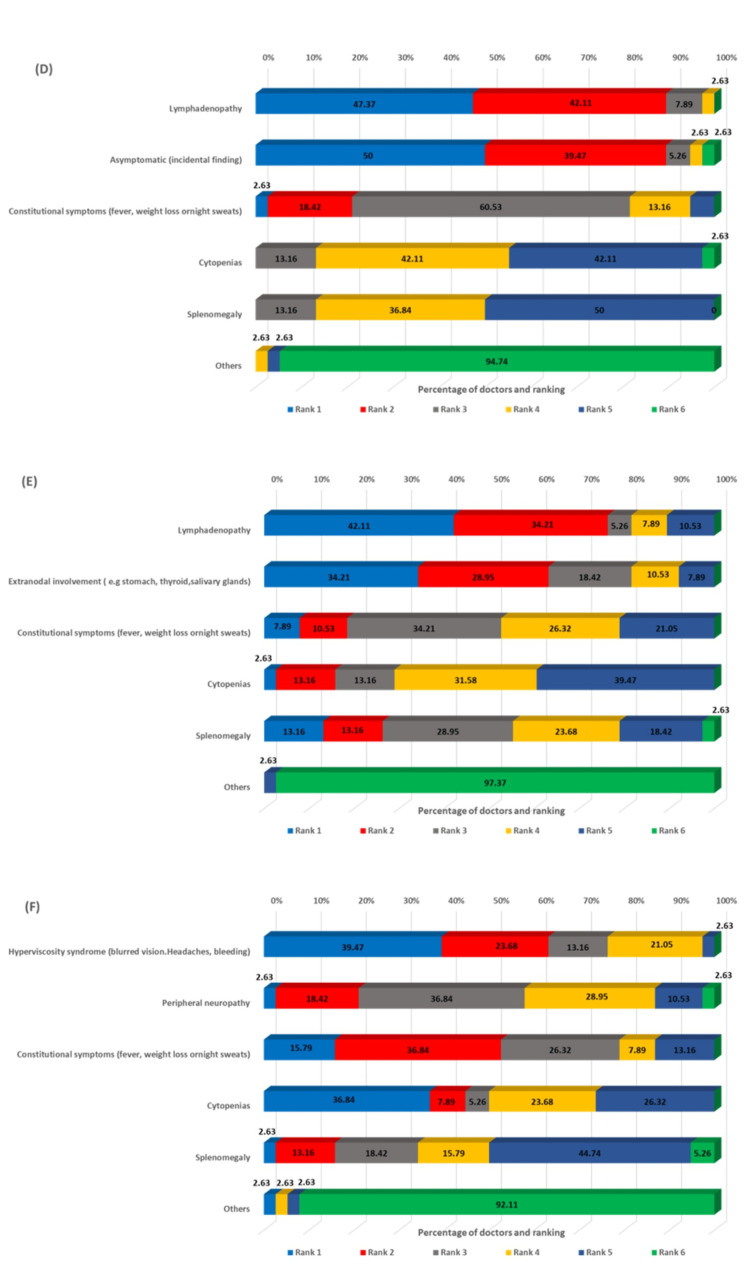
Ranking of clinical presentations from most common to least common in MZL (D), FL (E), and WM (F) MZL: marginal zone lymphoma, FL: follicular lymphoma, WM: Waldenström’s macroglobulinemia

The survey assessed the most common clinical presentations across different B-cell lymphoma subtypes. For DLBCL, rapidly enlarging lymphadenopathy was identified as the most common presentation (68.42% ranking it first), followed by constitutional symptoms and extranodal involvement (Figure 1A). In CLL, asymptomatic lymphadenopathy or lymphocytosis, as incidental findings, dominated the clinical presentation (89.47%), ranking first, reflecting the indolent nature of early-stage disease (Figure 1B). For MCL, lymphadenopathy was the predominant presentation (73.68%) (Figure 1C), whereas FL showed a more varied pattern, with lymphadenopathy (47.37%) and asymptomatic incidental findings (50.0%) equally common (Figure 2D). In MZL, lymphadenopathy (42.11%) and extranodal involvement (34.21%) were the most frequent presentations (Figure 2E), while WM characteristically presented with hyperviscosity syndrome (39.47%) (Figure 2F).

Patient demographics and molecular testing

Regarding the fitness of CLL patients at treatment initiation, the survey revealed that 34.21% of respondents indicated that 40-55% of their CLL patients were young or fit. In comparison, slightly more than a quarter (26.32%) reported less than 25% of their patients falling into this category.

Molecular testing practices showed high adoption rates for key prognostic markers. For newly diagnosed CLL patients, 63.16% of respondents were very likely to order TP53 testing, 71.06% to order del(17p) testing, and 68.42% to order IgHV mutation analysis. In WM patients, MYD88 testing was considered very likely by 68.42% of respondents, while CXCR4 testing showed more variable adoption (34.21% very likely) (Table 2).

**Table 2:** Molecular testing patterns in CLL and WM Responses were recorded using a 5-point Likert scale (1 = very unlikely, 2 = unlikely, 3 = sometimes, 4 = likely, 5 = very likely). CLL: chronic lymphocytic leukemia, CXCR4: C-X-C chemokine receptor 4, del(17p): deletion of the short arm of chromosome 17, IgHV: immunoglobulin heavy chain variable region, MYD88: myeloid differentiation primary response 88, TP53: tumor protein 53, WM: Waldenström macroglobulinemia

Disease	Molecular test	Very unlikely N (%)	Unlikely N (%)	Sometimes N (%)	Likely N (%)	Very likely N (%)
CLL	TP53	1 (2.63%)	-	6 (15.79%)	7 (18.42%)	24 (63.16%)
	del(17p)	3 (7.89%)	-	3 (7.89%)	5 (13.16%)	27 (71.06%)
	IgHV	1 (2.63%)	-	7 (18.42%)	5 (13.16%)	26 (68.42%)
WM	MYD88	2 (5.26%)	-	3 (7.89%)	7(18.42%)	26 (68.42%)
	CXCR4	3 (7.89%)	3 (7.89%)	10 (26.32%)	9 (23.68%)	13 (34.21%)

Treatment approach and BTKi usage

The wait-and-watch (active monitoring) approach showed notable variability: 47.37% of respondents reported using it in 50-75% of their CLL patients, while 21.05% each used it in more than 75% and 25-50% of their CLL patients, respectively. Eventually, most patients required treatment: 36.84% of respondents reported that 50-75% of their CLL patients, and 21.05% reported that 25-50%, ultimately received therapy, with 75-90% receiving treatment overall.

**Figure 3:**
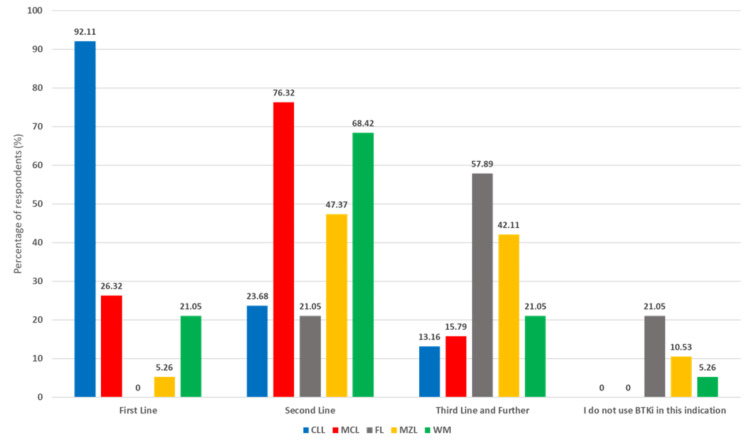
Usage of BTKis in B-cell lymphoma patients in practice BTKis: Bruton’s tyrosine kinase inhibitors, CLL: chronic lymphocytic leukemia, MCL: mantle cell lymphoma, FL: follicular lymphoma, MZL: marginal zone lymphoma, WM: Waldenström’s macroglobulinemia

BTKi use varied notably across lymphoma subtypes. In CLL, most respondents (92.1%) reported first-line use, reflecting strong evidence and guideline endorsement. In MCL, use was more heterogeneous, with the second-line setting being the most common (76.3%). In FL, BTKis were primarily reserved for later lines, predominantly third-line or beyond (57.9%). These trends align with the patterns illustrated in the graph below. MZL and WM showed intermediate patterns, with most usage occurring in second-line settings (Figure 3).

Treatment approaches and drug selection

Regarding treatment delivery for CLL, 63.16% of respondents preferred BTKis as monotherapy, while 21.05% used them in combination with chemoimmunotherapy, and 13.16% combined them with other targeted therapies. A striking finding was the dominance of generic acalabrutinib in clinical practice. While ibrutinib usage remained limited, with the majority (71.05%) of the hematologists prescribing it to <20% of eligible patients, generic acalabrutinib showed much higher adoption rates, with 42.11% of respondents prescribing it to almost all eligible patients and an additional 13.16% using it in 75-90% of cases. This preference pattern likely reflects both the improved safety profile of acalabrutinib compared to ibrutinib and cost considerations in the Indian healthcare setting.

Clinical decision-making factors

**Figure 4:**
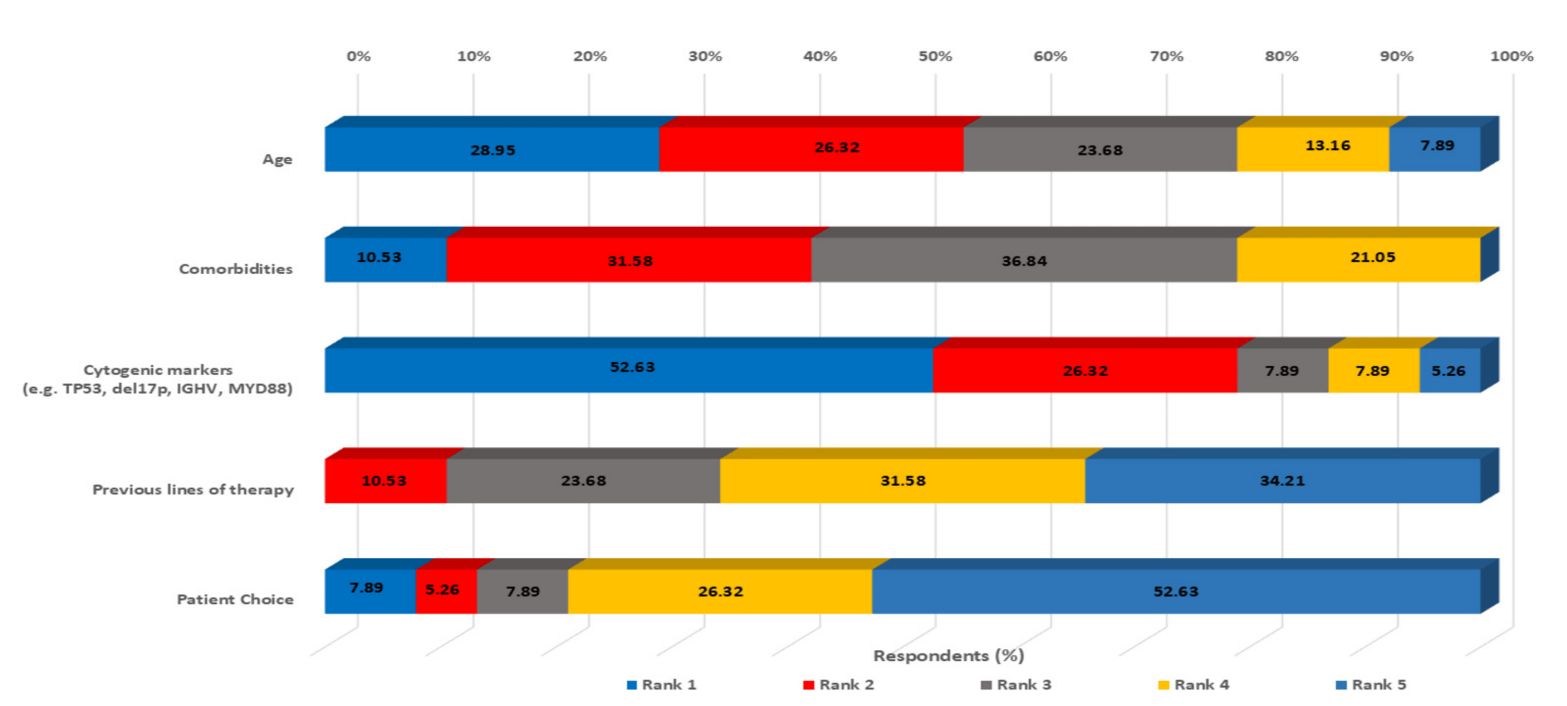
Ranking of the most important influential factors that affect the decision to initiate the BTKi del17p: deletion of the short arm of chromosome 17 (17p), IGHV: immunoglobulin heavy chain variable region, MYD88: myeloid differentiation primary response 88, TP53: tumor protein p53, BTKi: Bruton’s tyrosine kinase inhibitors

The survey identified cytogenetic markers as the most influential factor in BTKi initiation decisions (52.63% ranking it first), followed by age (28.95%) and comorbidities (10.53%). This emphasis on molecular markers reflects the growing understanding of their prognostic significance and their role in treatment selection. Previous lines of therapy and patient choice were ranked lower in terms of influence, suggesting that clinical and biological factors take precedence over other considerations in treatment decision-making (Figure 4).

Patient fitness and treatment selection

For elderly or frail patients with CLL or WM, 86.84% of respondents considered BTKis as the preferred treatment option compared to standard chemotherapy or chemoimmunotherapy. This strong preference reflects the established benefits of BTKis in older patients who may not tolerate intensive chemotherapy regimens. In contrast, for young or fit patients, only 39.47% considered BTKis universally preferred, with 57.89% reserving them for patients with poor prognostic biomarkers, such as del(17p) or unmutated IgHV in CLL, or MYD88 mutations in WM.

Discontinuation patterns and management

Treatment discontinuation patterns revealed important insights into real-world BTKi use. The most common reasons for discontinuation were intolerance or side effects (36.84%), followed by financial or access issues (34.21%), disease progression (23.68%), and patient non-compliance (5.26%). The high rate of discontinuation due to financial constraints highlights a significant challenge in the Indian healthcare context. For ibrutinib specifically, 39.47% of respondents reported discontinuation rates of 10-20% due to intolerance or side effects, while 26.32% reported rates of 30-40%. In contrast, acalabrutinib showed lower discontinuation rates: 26.32% of respondents reported 10-15%, and 23.68% reported 15-20%.

The most common side effects leading to ibrutinib discontinuation were cardiovascular events (78.95%), followed by bleeding (47.37%), gastrointestinal events such as diarrhea (31.58%), and infections (31.58%). (Note: Respondents were allowed to select multiple options; therefore, the percentages are not mutually exclusive and sum to more than 100%.) Musculoskeletal events, such as arthralgia (21.05%) and fatigue (18.45%), were other primary reasons for discontinuing ibrutinib. For acalabrutinib, cardiovascular concerns remained significant (39.47%), but musculoskeletal events (28.95%), gastrointestinal issues like diarrhea (28.95%), headache (23.68%), and rash (21.05%) were also prominent reasons.

Side effect management and switching patterns

**Figure 5:**
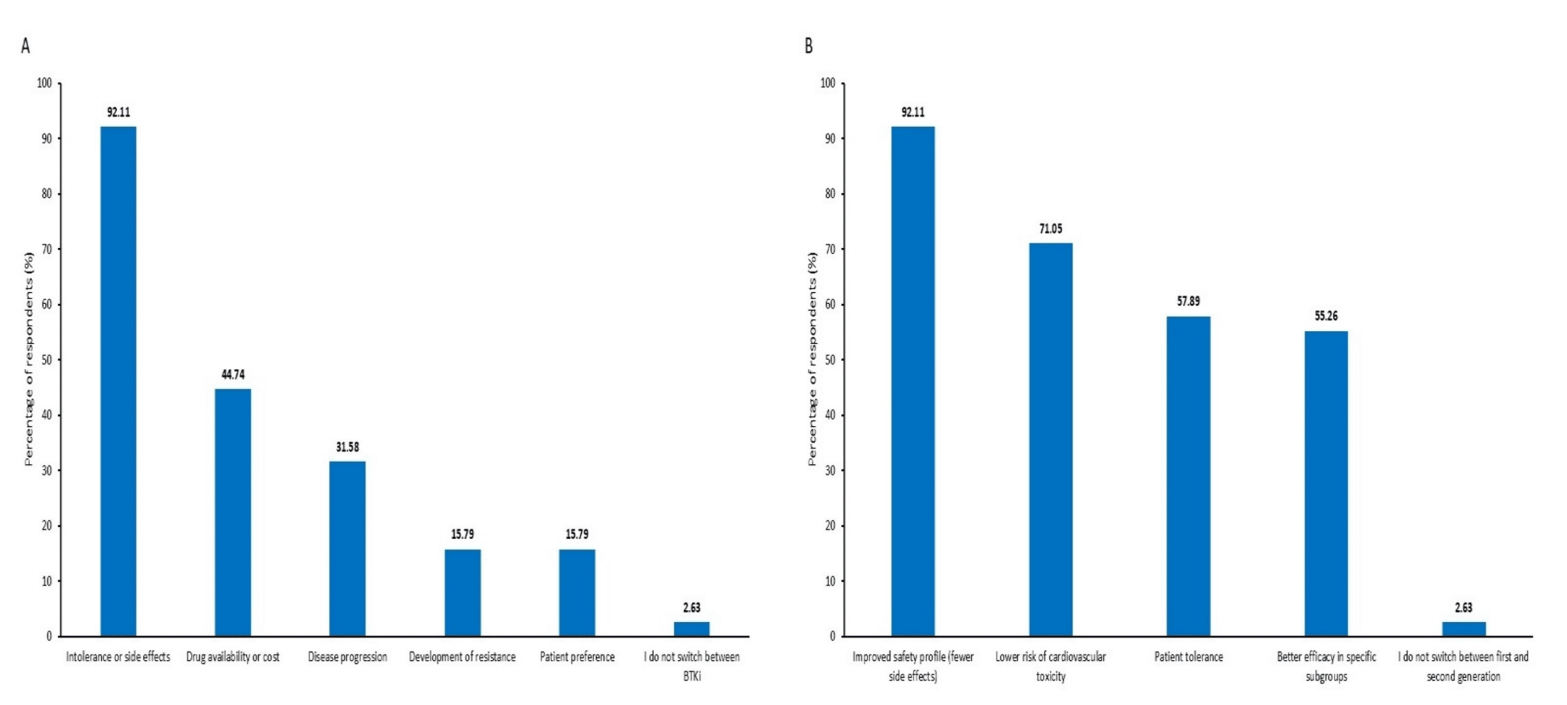
Factors guiding shift between BTKi and to second-generation BTKi from first-generation BTKi: Bruton’s tyrosine kinase inhibitor

Side-effect management strategies were comprehensive, with temporary discontinuation the most common approach (92.11%), followed by dose modification (71.05%) and symptomatic treatment (57.89%). Patient counselling was employed by 42.11% of respondents, emphasizing the importance of patient education in managing therapy. Switching between BTKis was primarily driven by intolerance or adverse effects (92.11%), followed by drug availability or cost considerations (44.74%), disease progression (31.58%), development of resistance (15.79%), and patient preference (15.79%). A small proportion of respondents reported not switching between BTKis (2.63%). The factors guiding switches to second-generation BTKis included improved safety profiles (92.11%), lower cardiovascular toxicity risk (71.05%), better patient tolerance (57.89%), better efficacy in specific subgroups (55.26%), and healthcare professionals' reluctance to switch between first- and second-generation (2.63%) (Figure 5).

Barriers and limitations

The survey identified significant barriers to the use of BTKis in the treatment of B-cell lymphoma. The high cost of therapy emerged as the predominant barrier, cited by 89.47% of respondents. This was followed by management of side effects (42.11%) and drug availability (36.84%). Other barriers included resistance development (13.16%), poor patient compliance (26.32%), indefinite duration of therapy (5.26%), and complex administration requirements (5.26%). These findings highlight the multifaceted challenges clinicians face in delivering optimal BTKi therapy. One of the respondents also highlighted that the non-inclusion of BTKi in the Ayushman Bharat scheme, India’s government-funded national health insurance program, represents a limitation on broader adoption and patient access. This finding underscores the broader policy-level challenges affecting equitable access to novel targeted therapies and highlights the need for alignment between clinical advances and public health reimbursement frameworks.

Unmet needs and future perspectives

Regarding unmet needs, improved access and availability were identified as the most pressing concern (68.42%), followed by resistance development and overcoming resistance mechanisms (55.26%). Looking ahead, respondents anticipated several key developments in BTKi therapy over the next five years. About 86.84% of respondents expected increased use in earlier lines of therapy, while 78.95% anticipated more combination approaches with other targeted therapies; 55.26% anticipated replacing chemotherapy, and 57.89% expected replacing covalent BTKis with non-covalent alternatives.

## Discussion

This survey, to our knowledge, provides a detailed first look at how BTKis are being utilized by hematologists in India and highlights both alignment with global standards and deviations driven by local challenges.

Consistent with published Indian registry data, DLBCL remains the most common B-cell lymphoma subtype encountered (40% of cases) [26]. CLL was relatively less common (22% of B-cell lymphoma cases), reflecting the known lower incidence of CLL in Asia than in the West. Notably, Western reports often cite CLL as 25-30% of adult leukemias [27]. FL accounted for 13% of B-cell lymphoma cases in this region, which aligns with findings from the developing world and is lower than the rates observed in developed countries, as highlighted in a review of 4539 cases conducted by the International Non-Hodgkin Lymphoma Classification Project [28]. Indian studies likewise note a lower FL incidence than in Western populations [29]. Mantle cell (8%) and marginal zone (6%) lymphomas were uncommon, as expected; for example, a large Indian case series reported MCL in ~4.6% of lymphomas and MZL subtypes in ~7% [30]. WM was rare overall, although our survey found that 5% of respondents managed patients with WM. This aligns with existing literature indicating that WM is an uncommon lymphoma (WM incidence is ~3-4/million per year globally) [31].

The clinical presentations reported in our survey closely reflected established patterns across B-cell lymphomas. DLBCL was characterized by rapidly progressive lymphadenopathy [32], CLL by incidental lymphocytosis or nodal enlargement [33], and FL by painless generalized lymphadenopathy [34]. MCL predominantly presented at advanced stages, with nodal and extranodal disease [35]; MZL frequently involved extranodal sites [36]; and WM was chiefly associated with hyperviscosity symptoms [37].

The age of CLL presentation in India, as indicated by 34.21% of respondents reporting that 40-55% of their patients were aged ≤55 years or were clinically fit (defined by good performance status and limited comorbidities), corroborates recent real-world evidence. Consistent with our survey findings, a single-center Indian study has also shown a relatively younger CLL population, reporting a median age of 60 years and 36.5% of patients aged ≤55 years [38]. This younger demographic pattern has important therapeutic implications, as evidenced by our finding that only 39.47% of respondents considered BTKi universally preferred for young patients, with most reserving it for those with poor prognostic biomarkers.

The reported adoption of molecular testing suggests increasing integration of prognostic markers into CLL care in India, with over 60% of respondents very likely to order key prognostic markers. The emphasis on TP53 testing (63.16%) and del(17p) analysis (71.06%) reflects growing recognition of their therapeutic implications. This is particularly relevant given that real-world data suggest TP53 aberrations may be present in higher proportions than previously recognized when using high-sensitivity methods [39]. The finding that cytogenetic markers ranked as the most influential factor (52.63%) in BTKi initiation decisions demonstrates evidence-based treatment selection, consistent with international guidelines [39-41]. In WM, the high uptake of MYD88 testing (68.42%) reflects its role as a key diagnostic and predictive marker for response to BTKis. In contrast, the more variable adoption of CXCR4 testing (34.21%) reflects its use primarily as a predictive tool to guide treatment selection rather than as a marker with independent prognostic value [42].

In CLL, nearly all respondents used BTKis as first-line therapy (92.11%), reflecting robust evidence and guideline endorsement of BTKi (ibrutinib, acalabrutinib, and zanubrutinib) as frontline options. Indeed, Spanish and National Comprehensive Cancer Network (NCCN) guidelines now prioritize targeted agents (BTKi or venetoclax combos) over chemoimmunotherapy for most CLL patients [40,43]. By contrast, MCL therapy in India still often begins with chemoimmunotherapy (e.g., bendamustine/rituximab or R-CHOP regimens), and BTKi are typically reserved for relapsed disease. For MCL, the varied usage pattern (26.32% first-line, 76.32% second-line) reflects ongoing evolution in treatment approaches and the heterogeneous nature of this disease [44]. Similarly, BTKi in FL were mainly used in third-line or beyond; this matches the fact that until recently, FL therapy centered on anti-CD20 antibodies and chemotherapy, and only now has zanubrutinib + obinutuzumab received accelerated approval in 2024 for relapsed FL after ≥2 prior therapies [45,46]. In MZL, BTKi were mostly second-line, consistent with their approved use only after failure of an initial anti-CD20 regimen. Ibrutinib was voluntarily withdrawn from the U.S. market for MZL in 2023, following an FDA consultation that noted confirmatory trials had not sufficiently verified its clinical benefit [12]. This action leaves zanubrutinib as the only FDA-approved BTKi for this specific patient population [47].

BTKi were strongly preferred in elderly or frail patients (>65 years), with 86.8% of respondents favoring them over chemotherapy for CLL and WM, reflecting superior tolerability in this population. In younger, fit patients (<65 years), BTKi were used more selectively, often reserved for high-risk features such as del(17p) or unmutated IGHV. This approach mirrors ongoing debates in the field: while chemo-free regimens are increasingly supported, the European Society for Medical Oncology (ESMO) endorses ibrutinib monotherapy for patients ineligible for chemotherapy [48], and NCCN favors chemotherapy-free options broadly; chemoimmunotherapy (e.g., FCR: fludarabine, cyclophosphamide, rituximab) remains a viable option for younger, good-risk patients, especially considering long-term data and cost implications of continuous BTKi therapy [43].

Notably, the availability of generic second-generation BTKi appears to have influenced prescribing practices in India. While ibrutinib prescriptions were low (over 70% of doctors used it in <20% of eligible patients), generic acalabrutinib was widely adopted (42.11% of doctors used it in nearly all eligible patients). This likely reflects acalabrutinib’s favorable safety profile (less cardiotoxicity) [17,49] and the recent availability of a generic, lower-cost version. A 2020 American Society of Clinical Oncology analysis noted that ibrutinib (140 mg) costs $4700 in India, compared with a generic priced at $410, which is a 90% reduction [50].

The survey data reinforce known differences in toxicity between ibrutinib and acalabrutinib. An extensive retrospective study showed toxicity was the dominant cause of ibrutinib discontinuation (>60% of stoppages), with arthralgia and atrial fibrillation being the most common [51]. Our respondents similarly reported cardiac issues (78.95%) and bleeding (47.37%) as the top ibrutinib side effects leading to cessation. For acalabrutinib, cardiotoxicity was much lower (39.47%), and other side effects like GI upset, headache, rash, and arthralgia predominated. These real-world impressions align with Phase II clinical trial findings, where acalabrutinib was discontinued due to headache, rash, arthralgia, and diarrhea [52]. Dose holds, or switches, were uniformly practiced; nearly all doctors would temporarily stop BTKi for adverse events, and 92.11% said toxicity drives switching between BTKi. Importantly, 92.11% prioritized switching to a second-generation BTKi (acalabrutinib or zanubrutinib) when intolerance occurs, valuing their lower cardiac risk and better tolerability [51,52]. These management patterns reflect the growing consensus that patients intolerant of ibrutinib can often continue on acalabrutinib and zanubrutinib [51,52], and that patients intolerant of acalabrutinib can continue on zanubrutinib [52].

The most significant obstacle reported was cost, cited by 89.47% of doctors, underscoring that even with generics, BTKi remains expensive for many Indian patients. The out-of-pocket expenses in India are well documented: the government PM-JAY scheme caps benefits (about $6000/year), which is often insufficient for high-priced novel therapies, and BTKi are not routinely covered under the PM-JAY scheme, further limiting access for patients without private insurance [53]. Physicians also noted drug availability and side effect management as barriers.

The anticipated developments over the next five years, including increased use in earlier lines (86.84%) and more combination approaches (78.95%), reflect global trends toward earlier intervention and novel therapeutic strategies. Combinations of BCL2 inhibitors with CD20 antibodies or BTKis have been investigated to develop time-limited targeted treatment strategies. Such finite therapies may reduce treatment-related toxicity and adverse effects. They could potentially reduce the risk of clonal evolution and resistance, as drug exposure is limited to a few cycles [53]. The expectation of replacing chemotherapy (55.26%) and transitioning to covalent and non-covalent BTKis (57.89%) suggests awareness of emerging therapeutic options [54]. However, these transitions will likely be influenced by cost and availability factors in the Indian context. The emphasis on improved access and availability as the most pressing unmet need (68.42%) underscores the critical importance of health policy initiatives and pharmaceutical pricing strategies to enable optimal patient care. The integration of targeted therapies into government insurance schemes and the expansion of generic manufacturing will be crucial for addressing these gaps [55].

Limitations

This study has several limitations that should be acknowledged. The use of convenience sampling may limit the generalizability of these findings to other healthcare settings. Future studies could address this limitation by employing more representative sampling approaches, such as stratified random sampling from national hematology or cancer registries, better to capture practice patterns across diverse regions and healthcare systems. The cross-sectional design provides a snapshot of current practices but cannot capture the dynamic evolution of treatment patterns over time. Additionally, the survey methodology relies on self-reported practices, which may not always reflect actual clinical decision-making in complex real-world scenarios and may be subject to social desirability bias, including potential over-reporting of guideline-recommended practices such as molecular testing. The response rate of 76%, while acceptable for survey research, means that the perspectives of non-responding physicians are not captured, potentially introducing selection bias. As the survey focused primarily on physician perspectives, it did not directly assess patient-reported outcomes or satisfaction with treatment approaches, which limits the interpretation of specific findings.

## Conclusions

This comprehensive survey of Indian hematologists reveals a rapidly evolving landscape of BTKi use in B-cell lymphomas, characterized by evidence-based treatment selection, appropriate risk stratification, and a sophisticated understanding of disease biology. The predominant use of BTKis as first-line therapy for CLL and the preference for second-generation agents reflect good adherence to current evidence and guidelines. The high discontinuation rates due to financial constraints underscore the need for healthcare policy interventions to improve drug accessibility and affordability. The survey findings highlight several areas for future focus: improving drug accessibility and affordability, developing strategies to overcome resistance mechanisms, optimizing combination approaches for time-limited therapy, and continuing education on side-effect management and treatment sequencing. The anticipated evolution toward non-covalent BTKis and combination approaches offers promise for addressing current limitations while maintaining or improving therapeutic outcomes. These insights provide valuable guidance for clinicians, researchers, and policymakers working to optimize BTKi therapy in B-cell lymphomas and highlight the importance of addressing healthcare system barriers to ensure equitable access to these transformative therapies.

## Appendices

Questionnaire

Mapping the BTKi landscape in B-cell lymphoma: a questionnaire-based perspective of Indian hematology experts

Section A: Demographics and Experience

1. Name:

2. Hospital name:

3. Years of experience as a practicing hematologist:

a. 0-5 yrs

b. 6-10 yrs

c. 11-15 yrs

d. >15 yrs

Section B: Practice patterns using BTKi

4. What is the incidence of the following B-cell lymphomas in your practice

**Table 3:** What is the incidence of following B-cell lymphomas in your practice?

Type of B-cell lymphoma	Percentages
Diffuse B-cell lymphoma (DLBCL)	
Chronic lymphocytic leukemia (CLL)	
Mantle cell lymphoma (MCL)	
Follicular lymphoma (FL)	
Marginal zone lymphoma (MZL)	
Waldenström macroglobulinemia (WM)	

5. Rank the following clinical presentation from most common to least common for each of the following lymphomas in your clinical practice

**Table 4:** Rank the following clinical presentation from most common to least common for each of the following lymphomas in your clinical practice

Type of B-cell lymphoma						
Diffuse B-cell lymphoma (DLBCL)	Rapidly enlarging lymphadenopathy	Extra nodal involvement (CNS, GI tract, Liver, bone marrow	Constitutional symptoms (fever, weight loss or night sweats)	Cytopenias	Bulky disease (large tumor masses)	Others
Chronic lymphocytic leukemia (CLL)	Asymptomatic lymphadenopathy/lymphocytosis OR Incidental finding	Symptomatic lymphadenopathy	Constitutional symptoms (fever, weight loss or night sweats)	Cytopenias	Splenomegaly	Others
Mantle cell lymphoma (MCL)	Lymphadenopathy	Gastrointestinal involvement (e.g., polyps)	Constitutional symptoms (fever, weight loss or night sweats)	Cytopenias	Splenomegaly	Others
Follicular lymphoma (FL)	Lymphadenopathy	Asymptomatic (incidental finding)	Constitutional symptoms (fever, weight loss or night sweats)	Cytopenias	Splenomegaly	Others
Marginal zone lymphoma (MZL)	Lymphadenopathy	Extranodal involvement (e.g., stomach, thyroid, salivary glands)	Constitutional symptoms (fever, weight loss or night sweats)	Cytopenias	Splenomegaly	Others
Waldenström macroglobulinemia (WM)	Hyperviscosity syndrome (blurred vision, headaches, bleeding)	Peripheral neuropathy	Constitutional symptoms (fever, weight loss or night sweats)	Cytopenias	Splenomegaly	Others

6. How likely are you to order following mutational testing in newly diagnosed CLL patients?

**Table 5:** How likely are you to order following mutational testing in newly diagnosed CLL patients? CLL: chronic lymphocytic leukemia

Type of mutational testing	Very unlikely	Unlikely	Sometimes	Likely	Very likely
TP53					
Del17p					
IgHV					

7. How likely are you to order following mutational testing in newly diagnosed WM patients?

**Table 6:** How likely are you to order following mutational testing in newly diagnosed WM patients? WM: Waldenström’s macroglobulinemia

Type of mutational testing	Very unlikely	Unlikely	Sometimes	Likely	Very likely
Myd88					
CXCR4					

8. What % of your CLL patients do you go for the wait and watch (active monitoring) approach?

a. <25%

b. 25-50%

c. 50-75%

d. >75%

9. What % of your CLL patients (including those previously on a wait-and-watch approach) eventually receive treatment?

a. >90%

b. 75-90%

c. 50-75%

d. 25-50%

e. <25%

10. How likely are you to use BTKis in following B-cell lymphoma patients in your practice?

**Table 7:** How likely are you to use BTKis in following B-cell lymphoma patients in your practice? BTKis: Bruton’s tyrosine kinase inhibitors

Type of B-cell lymphoma	Very unlikely	Unlikely	Sometimes	Likely	Very likely
Chronic lymphocytic leukemia (CLL)					
Mantle cell lymphoma (MCL)					
Follicular lymphoma (FL)					
Marginal zone lymphoma (MZL)					
Waldenström macroglobulinemia (WM)					

11. At what stage do you typically initiate BTKis in the following B-cell lymphomas? (mark all that applies)

**Table 8:** At what stage do you typically initiate BTKis in the following B-cell lymphomas? (mark all that applies) BTKis: Bruton’s tyrosine kinase inhibitors

Type of B-cell lymphoma	First line setting	Second line setting	Third line and further	I do not use BTKi in this indication
Chronic lymphocytic leukemia (CLL)				
Mantle cell lymphoma (MCL)				
Follicular lymphoma (FL)				
Marginal zone lymphoma (MZL)				
Waldenström macroglobulinemia (WM)				

12. Do you consider BTKi as a monotherapy or in combination with other treatments?

a. Primarily monotherapy

b. In combination with chemoimmunotherapy

c. In combination with other targeted therapies

d. Any other (please specify):

13. Rank the following factors from most influential to least that affect your decision to initiate the BTKi?

a. Age

b. Comorbidities

c. Cytogenic markers (e.g. TP53, del17p, IgHV, Myd88)

d. Previous lines of therapy

e. Patient choice

14. What % of your new patients eligible for BTKi are on ibrutinib?

a. <2%

b. 2-5%

c. 5-10%

d. 10-20%

e. Any other (please specify):

15. What % of your new patients eligible for BTKi are on generic acalabrutinib?

a. <50%

b. 50-60%

c. 60-75%

d. 75- 90%

e. Almost all

f. Any other (please specify):

16. For elderly or frail patients (esp. of CLL/WM), do you find BTKis to be the preferred treatment option compared to standard chemotherapy/chemoimmunotherapy?

a. Yes, in all patients

b. No

c. Only in patients with bad prognostic biomarkers (e.g., del17p and unmutated IgHV in CLL, myd88 in WM)

17. For young or fit patients (esp. of CLL/WM), do you find BTKis to be the preferred treatment option compared to standard chemotherapy/chemoimmunotherapy?

a. Yes, in all patients

b. No

c. Only in patients with bad prognostic biomarkers (e.g., del17p and unmutated IgHV in CLL, myd88 in WM)

18. What is the most common reason for discontinuation of BTKis in your patients?

a. Disease progression

b. Intolerance or side effects

c. Financial or access issues

d. Lack of efficacy

e. Patient non-compliance

f. Any other (please specify):

19. What percentage of your patients discontinue Ibrutinib therapy due to intolerance or side effects?

a. <10%

b. 10-20%

c. 20-30%

d. 30-40%

e. 40-50%

f. >50%

20. What percentage of your patients discontinue Ibrutinib therapy due to intolerance or side effects?

a. <5%

b. 5-10%

c. 10-15%

d. 15-20%

e. 20-25%

f. >25%

21. What is the most common side effect leading to discontinuation with Ibrutinib? (Mark all that applies)

a. Cardiovascular concerns (e.g., atrial fibrillation)

b. Infections

c. Bleeding

d. Fatigue

e. GI events (e.g., diarrhea)

f. Musculoskeletal events (e.g., arthralgia)

g. Any other (please specify):

22. What is the most common side effect leading to discontinuation with acalabrutinib? (Mark all that applies)

a. Headache

b. Cardiovascular concerns (e.g., atrial fibrillation)

c. Musculoskeletal events (e.g, arthralgia)

d. GI events (e.g. diarrhea)

e. Rash

f. Any other (please specify):

23. How do you typically manage BTKi-related side effects? (mark all that applies)

a. Dose modification

b. Temporary discontinuation

c. Permanent discontinuation

d. Symptomatic treatment (e.g., medications for side effects)

e. Counselling of the patient

f. Other (please specify):

24. In what circumstances do you consider switching from one BTKi to another? (mark all that applies)

a. Intolerance or side effects

b. Disease progression

c. Development of resistance

d. Drug availability or cost

e. Patient preference

f. Any other (please specify):

g. I do not switch between BTKi

25. What factors could guide your decision to switch to a second-generation BTKi (acalabrutinib, zanubrutinib) from first-generation Ibrutinib? (mark all that applies)

a. Improved safety profile (fewer side effects)

b. Better efficacy in specific subgroups

c. Patient tolerance

d. Lower risk of cardiovascular toxicity

e. Other (please specify)

f. I do not switch between first and second generation

26. What are the main limitations or barriers to using BTKis in B-cell lymphoma treatment? (mark all that applies)

a. High cost of therapy

b. Availability of the drug

c. Management of side effects

d. Resistance development

e. Poor patient compliance

f. Complex administration and monitoring requirements

g. Other (please specify):

27. What do you consider the most significant unmet need regarding the use of BTKis in B-cell lymphoma? (mark all that applies)

a. Resistance development/overcoming resistance

b. Improved access and availability

c. More robust data in specific patient subgroups

d. Any other (please specify):

28. How do you foresee the role of BTKis evolving in the next five years for B-cell lymphoma?

a. Increased use in earlier lines of therapy

b. Combination with other targeted therapies (especially finite treatment duration)

c. Replacement of chemotherapy

d. Replacement of covalent BTKi with non-covalent BTKi

e. Any other (please specify):

Credits are given to Azra Naseem, Amullya Pednekar, Saiprasad Patil, and Hanmant Barkate.
